# Short-term evaluation of immediately-treated patients with acute HIV infection, recently diagnosed in the National Institute for Infectious Diseases "Prof. Dr. Matei Balş", Bucharest, Romania

**DOI:** 10.1186/1471-2334-14-S7-O1

**Published:** 2014-10-15

**Authors:** Ruxandra Moroti, Adriana Hristea, Violeta Molagic, Raluca Jipa, Mihaela Iosipenco, Doina Rîciu, Dan Oțelea, Dragoş Florea, Valeriu Gheorghiță, Raluca Hrişcă, Ioan Diaconu, Adrian Streinu-Cercel, Otilia Benea

**Affiliations:** 1 Carol Davila University of Medicine and Pharmacy, Bucharest, Romania; 2 National Institute for Infectious Diseases "Prof. Dr. Matei Balş", Bucharest, Romania; 3 Central Universitary Emergency Military Hospital Dr Carol Davila, Bucharest, Romania

## Background

The rapidly put-on-treatment in acute HIV infection (AHI) seems to achieve functional cure in up to 15% cases. This represents a huge difference, spontaneous elite-controllers being less than 0.5%.

Objective: To identify the AHI patients and to observe the immune-virological course under immediately-started antiretroviral treatment (ART).

## Methods

All newly-diagnosed HIV-infected adults (>18 yo) in the last 18 months (01.2013-06.2014) in an infectious diseases hospital were considered. The including criteria for AHI group were: detectable HIV-RNA or positive antigen/antibody combination assays in the setting of a negative/indeterminate HIV Western blot. AHI group was classified accordingly to Fiebig stages and was further evaluated regarding CD4 count and viral load (VL) at diagnosis, at 3 and 6 months. ART initiation and the regimen were also registered.

## Results

804 adults were newly-diagnosed HIV-positive, out of which 26 patients (2.32%) with AHI. The number of patients in Fiebig II/III, IV, V and VI stages was 8, 15, 2 and 1 respectively. The AHI-group had a median age of 31, IQR [25-34] and 3.1:1 male:female ratio. The median CD4 count was 435, IQR [251-775] and the median VL was 5.6 log10, IQR [4.7-7]. Eight out of 26 AHI patients immediately started ART, in Fiebig II/III and IV stages for 7 of them. The 8^th^ received ARV treatment in eclipse phase (for 28 days, as post-exposure prophylaxis) then restarted ART in Fiebig VI stage, at diagnosis moment. The immediately-treated group had a median age of 24, IQR [20-29], a male:female ratio of 7:1 and all were symptomatic. The median CD4 count at diagnosis was 261, IQR [147-467] and the median VL was 7 log10, IQR [5.6-7], except the partially-treated in eclipse-phase patient, whose CD4 count was 789 and VL was 21977c/mL at diagnosis. In the immediately-treated group there was a rise in median CD4 count to 646, IQR [544-764] at month 3 and to 755, IQR [577-950] at month 6. The median VL declines to 1.6 log10, IQR [1.3-2.2] at month 6. Five patients received a 3-drugs regimen and 3 received a 4-drugs regimen. The immune-virological course couldn’t be correlated with a particular regimen or with the number of drugs used.

## Conclusion

Two percent of newly diagnosed HIV-infected patient in 18 months in our setting had AHI and one third of them received immediate treatment. The short-term benefit was the consistent immune-virological improvement, regardless of ART scheme. However, more than half had detectable VL at month 6, probable due to the very high initial VL.

